# "The Tribune" Exhibition of Winter Foods and Drinks

**Published:** 1908-01-25

**Authors:** 


### "THE TRIBUNE" EXHIBITION OF WINTER FOODS AND DRINKS.

Following the Exhibition of Summer Foods and Drinks which was held last July, *The Tribune* has organised a Winter Exhibition of the same kind. This was opened by Lieut.-Colonel Newnham-Davis on Monday afternoon, January 20th, at *The Tribune* Rendezvous in Bouverie Street, and continues open during the remaining afternoons of this week. On Tuesday there was a good attendance of visitors, who all appeared much interested.

Amidst a bewildering assortment of strange new foods and still stranger beverages, chiefly derived from the vegetable kingdom, we noticed a number of exhibits of standard dietetic preparations and appliances, and of novelties recently issued by well-known firms of manufacturers. Considerations of space forbid us to do more than briefly draw attention to the special features of the more important of these latter exhibits.

Messrs. Allen and Hanburys, Ltd., have on show, in addition to their more familiar preparation, numerous samples of the Allenbury Milk Food Chocolate, Pancreatised Milk Cocoa, and Concentrated Liquid Beef. Bovril, Ltd., are exhibiting a showcase containing bottles of Bovril of various sizes and a selection of Bovril Lozenges and Tablets. Another substance of good repute in the eyes of the medical profession—namely, Cerebos Salt—is represented by a large collection of packages and salt-bottles. Messrs. J. S. Fry and Sons, besides their Cocoa and Milk Chocolate, have on show a selection of tins of their Malted Cocoa, a valuable preparation issued by them and Messrs. Allen and Hanburys. Among the specialities for invalids displayed by Messrs. Brand and Co. we noticed especially their "home-made" Concentrated Beef-Tea, Savoury Meat Lozenges, and Beef-Tea Tabules, and their Invalid Soups prepared without fat. Plasmon, Ltd., make a special point in their exhibit of a variety of new Plasmon Biscuits and Plasmon loaves which are pleasant to taste, and of Plasmon Oats for the rapid preparation of nutritious porridge. The Marmite Food Extract Co., Ltd., are showing specimens of Marmite, which is a vegetable extract made from yeast-cells —very like meat-extract in character and composition, but considerably cheaper. They also show Marmite ("Savoy" Brand), a recent refinement of their original extract. Messrs. Brown and Polson's standard Cornflour and Paisley Flour are on view upon one of the stalls, and visitors are offered excellent little cakes, etc., made with these substances. Messrs. Keen Robinson and Co., whose Barley is largely recommended by the medical profession for making barley water, is also represented.

Among the various cooking and heating apparatus we noticed a large display of Wellbank's Boilerettes of different sizes and shapes. This simple and efficient appliance cooks the food within a water-jacket, which is supplied with a safety-valve. In this group of exhibits we were much impressed by the new patent "Eetot" Cooker, manufactured by Messrs. A. E. Gutmann and Co., who have already introduced with such success the "Thermos" Flask, which is also on show. The "Eetot" Cooker should be of service to night nurses and others who have to cook food and cannot spare time for its supervision. Finally, we may say that of the various cups of coffee which we tasted at different stalls, that prepared from Messrs. Rapson's Coffee contrasted favourably with the ingenious substitutes and adaptations of the many "food reform" exhibitors.

